# Return To Play Rate and Performance Following Surgical Repair of Athletic Pubalgia in Major League Soccer Players: A Retrospective Case-Control Study

**DOI:** 10.7759/cureus.38023

**Published:** 2023-04-23

**Authors:** Briley Guarneri, Logan Morrison, Adam Martorana, Ishan Gujral, Lafe Harris

**Affiliations:** 1 School of Osteopathic Medicine Arizona, A.T. Still University, Mesa, USA

**Keywords:** major league soccer, soccer, performance, sports hernia, athletic pubalgia

## Abstract

Introduction

Athletic pubalgia (AP) injuries requiring surgical repair in elite-level soccer players are significant injuries with the potential of impacting a player's playing time and performance. Currently, no data exists explicitly analyzing Major League Soccer (MLS) players' return to play (RTP) rates and performance following these surgeries.

Methods

A retrospective review of publicly available data of all MLS players who underwent surgery to repair an isolated AP injury from the league inception year of 1993 through 2021 was performed. Demographic data at the time of injury was collected. Athletes who successfully returned to play for at least two seasons in the MLS were matched to healthy controls in a 1:2 ratio by demographics and position. The index year was defined as the season, including pre- and post-season, that the surgery occurred. RTP date and performance metrics one and two years pre- and post-index year were collected. Statistical analysis was performed.

Results

Eighty-eight players underwent surgical repair for AP from 1993 through 2021. Eighty-five athletes were able to successfully RTP (96.5%). Twenty-five players met the inclusion criteria and were included in the final analysis. The average RTP time was 1.08±4.92 months. During the combined seasons following surgery, athletes in the AP group displayed a significant reduction in minutes played compared to the two combined seasons prior to surgery (4153±912.77 vs. 3405.36±1342.35 minutes; p=0.03). There was no significant reduction in performance metrics when compared to both prior season statistics and the matched cohort (p>0.05).

Conclusion

There is a high RTP rate among MLS players who undergo isolated surgical repair of AP. Although there was a significant reduction in combined minutes played in the two ensuing seasons following surgery, athletes who RTP demonstrated equivalent performance metrics comparable to their pre-injury seasons as well as to a matched cohort.

## Introduction

Injuries involving the lower extremity, groin, and pelvis have been shown to comprise nearly 80% of all injuries in Major League Soccer (MLS) players [1]. Groin pain in the past has been a difficult diagnosis to elucidate due to the anatomical complexity of the region and the oftentimes variable pain pattern amongst injured athletes [2,3]. The incidence of athletic pubalgia (AP) has grown in its recognition as a common source of both acute and chronic groin pain [2-5]. Over the years, there have been many different names used to define AP, such as Gilmore's groin, sports hernia, sportsmen's groin, footballers groin injury complex, ﻿osteitis pubis, hockey player's syndrome, and athletic hernia [1,3,6]. AP, by definition, is an inflammatory condition of the pubic symphysis caused by a deficiency of the posterior wall of the inguinal canal [2,7]. Pain is often elicited upon the activity or deep palpation at the fascial attachments of the rectus abdominis and adductors onto the pubis [2,6,7]. Treatment for AP remains predominantly athlete-level dependent. First-line treatment commonly consists of a period of rest and anti-inflammatory medication [8]. Once conservative therapy has failed, there are surgical fixation techniques readily available for use [7-9].

Soccer players are at increased risk for groin injuries and AP due to the sport's requirement of a high amount of pivoting and quick changes of direction along with constant acceleration, deceleration, and kicking [2,5]. In a retrospective study evaluating 8490 athletes that had been diagnosed with AP, 44.6% were soccer players, with the next closest sport comprising only 22.3% of the study population [6].

A previous systematic review done by Serafim et al. found that athletes who underwent surgery for AP returned to sport earlier than those receiving conservative treatment alone [10]. This may explain why elite-level athletes, in comparison with their recreational counterparts, may opt for earlier surgical fixation following a diagnosis of AP. Currently, no data exists evaluating the impact surgical intervention of AP has on MLS players. The purpose of this study was to evaluate the effect of surgical fixation for AP in MLS players by determining return to play (RTP) rates and performance metrics following surgery.

## Materials and methods

A retrospective review of all publicly available data of MLS players who underwent surgery to repair an isolated AP injury from the league inception year of 1993 through 2021 was performed. Due to the public nature of the data, IRB approval was not needed. Similar to previous studies evaluating RTP statistics in professional athletics, available injury reports, team news articles, individual player timelines, and game statistics were accessed through affiliated team websites (mlssoccer.com and transfermarkt.us) [11-13]. Two separate injury reports or articles were considered sufficient for player inclusion in preliminary data collection. To ensure the accuracy of the injury reports, concurrent game statistics for the reported time the player received surgery were checked to confirm the player's game absence.

**Figure 1:**
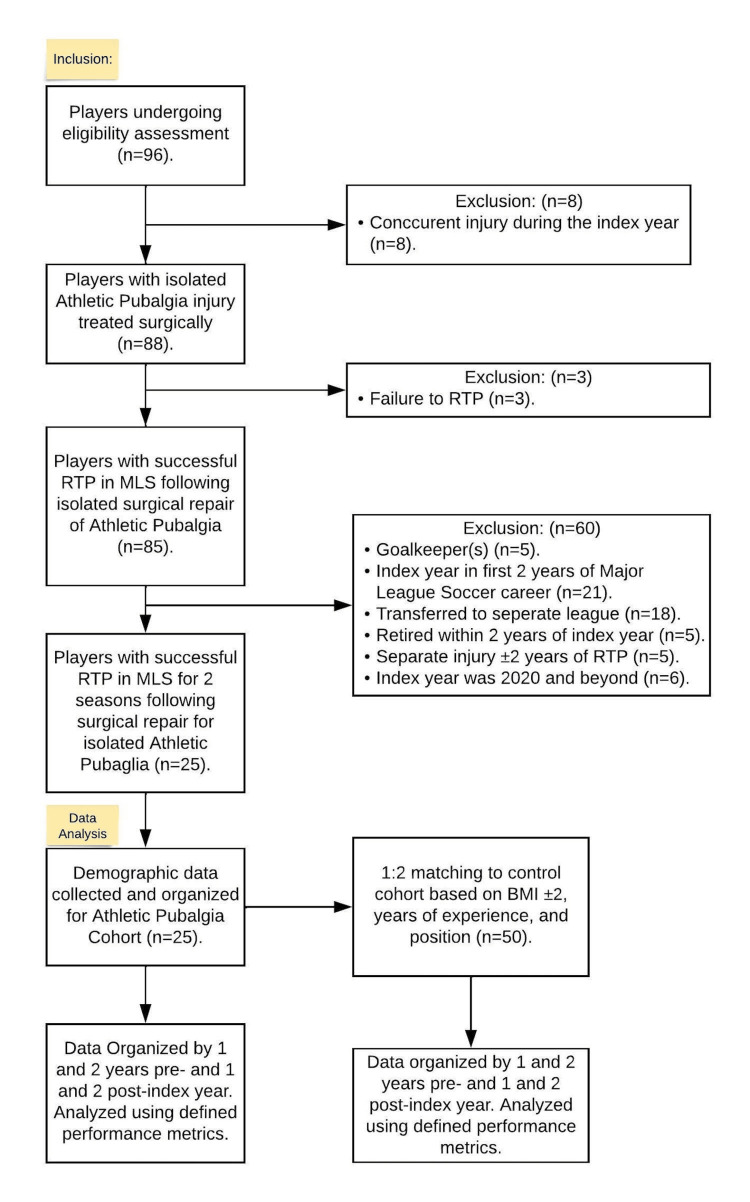
Subject inclusion flow chart MLS - Major League Soccer; RTP - return to play

Athletes were excluded from the final data analysis if they failed to RTP, had concurrent injuries at the time of AP surgery, suffered a significant injury within one year of their RTP date, or if the player left the MLS within one year (Figure 1). Goalkeepers were also excluded from the study as the position does not record statistics that can be accurately compared to outfield players. To be included in the study, the player must have played at least two years in the MLS, both prior to their surgery as well as after. The index year was defined as the season, including pre- and post-season, that the surgery occurred. Age, position, and body mass index (BMI) were collected for each athlete.

The study group was matched to healthy controls in a 1:2 ratio using age, weight, height, BMI, and MLS experience. ﻿A priority was given to matching age and position amongst the cohorts to alleviate differences in league era, years of experience, and statistical discrepancies. The index year for control players was chosen as the injury year of the study group match. RTP date and performance metrics, which included matches, minutes, shots, shots on goal (SOG), assists, and goals, one and two years pre- and post-index year, were collected for both study groups.

Statistical analysis was performed to compare data before and after the index year for both the control and study groups. The data was analyzed using SPSS software (IBM Inc., Armonk, US). All variables were checked for normality, and testing was used accordingly. For those that followed a normal distribution, a t-test was used; otherwise, a Wilcoxon rank sum test was used. An independent samples t-test was used to compare the statistical means between both groups. Pearson's correlation coefficient was used to examine correlations between each demographic characteristic and RTP.

## Results

Demographic data

Eighty-eight players underwent surgical repair for AP from 1993 through 2021. Eighty-five athletes were able to successfully RTP (96.5%). Only twenty-five MLS players met the inclusion criteria and were subsequently matched to healthy controls, with all twenty-five players successfully returning to play. There were no significant differences between the AP group and controls regarding demographic data that was collected (Tables 1 and 2). The average RTP was 1.08±4.92 months.

**Table 1:** Correlations of demographic characteristics with RTP RTP - return to play

Characteristics	r	p-value
Age	0.051	0.810
BMI	0.106	0.615
Experience	0.221	0.288
Position	0.236	0.256
Height	0.293	0.155
Weight	0.306	0.137

**Table 2:** Population demographics at the time of injury RTP - return to play

Characteristics	Athletic pubalgia (n=25)	Controls (n=50)	p-value
Position	n (%)	n (%)	1
Defender	5 (20.0)	10 (20.0)	
Forward	8 (32.0)	16 (32.0)	
Midfielder	12 (48.0)	24 (48.0)	
	Mean (SD)	Mean (SD)	
Age (years)	27.16 (3.45)	27.44 (2.95)	0.715
Height (cm)	179.32 (4.97)	179.83 (6.20)	0.723
Weight (kg)	75.95 (5.06)	76.21 (6.14)	0.854
BMI (kg/m^2^)	23.61 (1.10)	23.56 (1.31)	0.859
Experience (years)	5.52 (2.60)	5.30 (2.68)	0.736
RTP (months)	1.08 (4.92)		

Game statistics

When comparing the combined statistics for one and two years pre-injury to one and two years post-injury, there was a significant difference in the minutes played for the AP group (4153±912.77 minutes versus 3405.36±1342.34; p=0.03) (Table 3). The AP group also showed a significant decrease in minutes (2119.48±509.72 versus 1547.04±842.96; p=0.01) and SOG (17.48±15.72 versus 9.12±10.46; p=0.04) when comparing data from one year pre-injury to two years post-injury (Table 4).

**Table 3:** Performance metrics combined one and two years pre- versus one and two years post-index ﻿Variables are given as mean (SD) for performance metrics between the AP and control groups with respective p-values. Significant values are p<0.05. AP - athletic pubalgia; SOG - shots on goal; y - years; matches - matches played; minutes - minutes played

Performance metrics		Athletic pubalgia (n=25)		Controls (n=50)	
		Mean (SD)	p-value	Mean (SD)	p-value
Matches	y 1 and 2 pre	52.64 (8.95)	0.14	51 (9.95)	0.09
	y 1 and 2 post	46.72 (12.84)		47.64 (9.60)	
Minutes	y 1 and 2 pre	4153 (912.77)	0.03	3839.88 (1105.91)	0.16
	y 1 and 2 post	3405.36 (1342.35)		3533.74 (1076.23)	
Goals	y 1 and 2 pre	9.88 (10.16)	0.47	6.7 (6.53)	0.91
	y 1 and 2 post	6.64 (5.82)		7.16 (8.50)	
Assists	y 1 and 2 pre	7.32 (4.89)	0.14	6.46 (5.47)	0.09
	y 1 and 2 post	5.4 (4.83)		4.7 (4.09)	
Shots	y 1 and 2 pre	79.32 (63.20)	0.15	61.2 (43.78)	0.29
	y 1 and 2 post	50.52 (40.43)		54.5 (47.07)	
SOG	y 1 and 2 pre	33.92 (30.14)	0.10	23.9 (19.29)	0.25
	y 1 and 2 post	20.16 (16.77)		20.16 (20.78)	

**Table 4:** Performance metrics at one year pre-index versus two years post-index Variables are given as mean (SD) for performance metrics between the AP and control groups with respective p-values. Significant values are p<0.05. AP - athletic pubalgia; SOG - shots on goal; y - years; matches - matches played; minutes - minutes played

Performance metrics		Athletic pubalgia (n=25)		Controls (n=50)	
		Mean (SD)	p-values	Mean (SD)	p-values
Assists	1 y pre	3.8 (2.87)	0.07	3.52 (3.67)	0.03
	2 y post	2.36 (2.41)		1.98 (1.97)	
Goals	1 y pre	4.72 (5.34)	0.19	3.68 (4.11)	0.45
	2 y post	2.84 (3.39)		3.42 (5.08)	
Matches	1 y pre	27.04 (4.21)	0.07	26.14 (5.48)	0.02
	2 y post	22 (9.38)		23.36 (5.99)	
Minutes	1 y pre	2119.48 (509.72)	0.01	1957.8 (628.89)	0.06
	2 y post	1547.04 (842.96)		1721.64 (625.44)	
Shots	1 y pre	39.56 (31.5)	0.08	33.86 (27.06)	0.08
	2 y post	23.96 (24.61)		26.24 (27.43)	
SOG	1 y pre	17.48 (15.72)	0.04	13.28 (11.43)	0.05
	2 y post	9.12 (10.46)		9.52 (11.53)	

There were no significant differences between the AP group and controls in matches, minutes, goals, assists, shots, and SOG (all p-values >0.05) when comparing data from both pre- and post-injury (Tables 5 and 6).

**Table 5:** Performance metrics at two and one years pre-index Variables are given as mean (SD) for performance metrics between the AP and control groups with respective P values. Significant values are p<0.05. AP - athletic pubalgia; SOG - shots on goal; y - years; matches - matches played; minutes - minutes played

Performance metrics	Athletic pubalgia (n=25)	Controls (n=50)	
				Mean (SD)	Mean (SD)	p-value
	Matches 2 y pre	25.60 (7.27)	24.86 (6.97)	0.67
Minutes 2 y pre	2033.52 (691.80)	1882.08 (703.54)	0.38
Goals 2 y pre	4.16 (4.24)	3.02 (3.70)	0.06
Assists 2 y pre	3.52 (2.87)	2.94 (2.77)	0.4
Shots 2 y pre	39.76 (32.46)	27.34 (22.22)	0.06
SOG 2 y pre	16.44 (15.01)	10.62 (10.46)	0.05
Matches 1 y pre	27.04 (4.21)	26.14 (5.48)	0.47
Minutes 1 y pre	2119.48 (509.72)	1957.80 (628.89)	0.27
Goals 1 y pre	4.72 (5.34)	3.68 (4.11)	0.35
Assists 1 y pre	3.80 (2.87)	3.52 (3.67)	0.74
Shots 1 y pre	39.56 (31.50)	33.86 (27.06)	0.42
SOG 1 y pre	17.48 (15.72)	13.28 (11.43)	0.19

**Table 6:** Performance metrics at one and two years post-index Variables are given as mean (SD) for performance metrics between the AP and control groups with respective p-values. Significant values are p<0.05. AP - athletic pubalgia; SOG - shots on goal; y - years; matches - matches played; minutes - minutes played

Performance metrics	Athletic pubalgia (n=25)	Controls (n=50)	
				Mean (SD)	Mean (SD)	P Value
	Matches 1 y post	24.28 (6.05)	24.72 (6.46)	0.77
Minutes 1 y post	1812.10 (633.29)	1858.32 (690.00)	0.77
Goals 1 y post	3.74 (4.11)	3.80 (3.50)	0.95
Assists 1 y post	2.72 (2.70)	3.04 (2.95)	0.64
Shots 1 y post	28.26 (22.72)	26.56 (18.59)	0.75
SOG 1 y post	10.64 (10.51)	11.04 (8.39)	0.87
Matches 2 y post	23.36 (5.99)	22.00 (9.38)	0.45
Minutes 2 y post	1721.64 (625.44)	1547.04 (842.96)	0.32
Goals 2 y post	3.42 (5.08)	2.84 (3.39)	0.61
Assists 2 y post	1.98 (1.97)	2.36 (2.41)	0.47
Shots 2 y post	26.24 (27.43)	23.96 (24.61)	0.73
SOG 2 y post	9.52 (11.53)	9.12 (10.46)	0.88

## Discussion

Athletic pubalgia, as a common source of chronic groin pain in high-level athletes, continues to rise in incidence. Accompanied by a difficult diagnosis is an oftentimes more difficult decision regarding treatment modality. Numerous surgical techniques, including open repair and tenotomy of muscles attached to the pubic bone as well as less-invasive laparoscopic techniques exist [6]. There is limited data today that supports one technique over another, and it is beyond the scope of this study to compare specific repair types to one another. There is, however, one study that has found endoscopic mesh repair offering athletes a faster recovery for AP in comparison to nonoperative therapy [7]. In this study, Paajanen et al. compared recovery times and pain ratings for 30 athletes who received surgical repair for AP and 30 patients receiving only conservative physiotherapy for AP. The laparoscopic repair cohort showed a one-month RTP rate of 67% in comparison to the 20% one-month RTP rate among the conservative treatment-only group [7].

With the MLS recently reporting record-setting years in both viewership and revenue for the previous few seasons and with the rapid expansion of soccer in the United States, the financial incentive for players to remain healthy has increased [14]. Due to the high incidence of AP occurring in MLS players, there needs to be specific data to guide the paradigm for the treatment of AP once a diagnosis has been made. This study aims to provide that by objectively defining performance parameters that are affected by previous surgical repair of AP.

Although surgery should most often be considered once conservative treatment has failed, our study adds to the current literature potentially supporting earlier surgical intervention as a viable option in high-level athletes who rely upon their sport as their primary source of income [2,6,7,11,15,16]. The results of this study identified a high RTP rate among MLS players (96.5%) who underwent isolated surgical repair of AP. Additionally, this study found that athletes who returned to play exhibited comparable performance metrics to both their pre-injury seasons as well as a matched cohort of healthy athletes during the same time frame. Previous studies have identified similar outcomes among other professional sports leagues in the United States and around the world. Castle et al. evaluated thirty-three National Basketball Association players that had undergone AP surgery and found that 90.91% (30/33) of them successfully returned to play in an average time of 4.73 months [11]. Piozzi et al. found that out of 198 soccer athletes, the RTP was 98.5%, with the cohort averaging an RTP time of four weeks [15].

Limitations

It is important to note that the study is not without limitations. Due to obtaining retrospective data that was publicly available, details such as specific surgical technique and the diagnostic criteria used were not readily available for collection. Additionally, public data retrieval is not always accurate when being reported through third-party sources. Another limitation of the study design was the exclusion criteria. The study population excluded players who had successfully returned to play but did not remain in the MLS for two full seasons following their surgery. With a high turnover rate amongst rosters, this made the sample size shrink considerably after initial data collection. Lastly, with players all coming from separate teams, access to different rehabilitation facilities and staff can influence outcomes regarding the RTP timeline. Future research in this area should focus on determining superior surgical techniques and rehabilitation protocols, specific physical exam findings that correlate with imaging findings, and a standardized paradigm for return to play in high-level athletes. Finally, subsequent research studies should include female professional sports leagues to determine if there is a significant difference in metrics based on sex. 

## Conclusions

The results of the study demonstrate a high RTP rate and equivalent performance compared to pre-injury seasons. When matched to healthy controls in a 1:2 ratio, the AP group consistently demonstrated performance parameters that were equal to their healthy counterparts. Although players in the AP group displayed reduced minutes played in the combined years following surgery, their game efficiency measured through statistics did not indicate a drop in their level of play. Based on this data, MLS players requiring surgery to repair an isolated AP injury should feel confident in returning to their baseline level of performance following their recovery. In addition, due to the demand of the sport and the financial impact of missing time playing, early surgical repair for isolated AP injuries may be an effective treatment for elite-level soccer players.
